# Mining Digital Traces of Facebook Activity for the Prediction of Individual Differences in Tendencies Toward Social Networks Use Disorder: A Machine Learning Approach

**DOI:** 10.3389/fpsyg.2022.830120

**Published:** 2022-03-08

**Authors:** Davide Marengo, Christian Montag, Alessandro Mignogna, Michele Settanni

**Affiliations:** ^1^Department of Psychology, University of Turin, Turin, Italy; ^2^Department of Molecular Psychology, Institute of Psychology and Education, Ulm University, Ulm, Germany

**Keywords:** digital footprints, digital phenotyping, problematic social media use, social networks use disorder, behavioral addictions, data mining

## Abstract

More than three billion users are currently on one of Meta’s online platforms with Facebook being still their most prominent social media service. It is well known that Facebook has designed a highly immersive social media service with the aim to prolong online time of its users, as this results in more digital footprints to be studied and monetized (*via* psychological targeting). In this context, it is debated if social media platforms can elicit addictive behaviors. In the present work, we demonstrate in *N* = 1,094 users that it is possible to predict from digital footprints of the Facebook users their self-reported addictive tendencies toward social media (*R* > 0.30) by applying machine-learning strategies. More specifically, we analyzed the predictive power of a set of models based on different sets of features extracted from digital traces, namely posting activity, language use, and page Likes. To maximize the predictive power of the models, we used an ensemble of linear and non-linear prediction algorithms. This work showed also sufficient accuracy rates (AUC above 0.70) in distinguishing between disordered and non-disordered social media users. In sum, individual differences in tendencies toward “social networks use disorder” can be inferred from digital traces left on the social media platform Facebook. Please note that the present work is limited by its cross-sectional design.

## Introduction

Spending time on social media is an increasingly popular online activity among both young and older adults, allowing them to interact with peers, meet new people, maintain relationships, promote one’s own image, as well as look out for news and entertainment (Sun and Zhang, 2021). The popularity of social media is in part due to their availability and easiness of use on mobile devices; amongst the most popular social media applications are Facebook, Instagram, YouTube, and TikTok (Statista, 2022). Despite the growing competition, to this day Facebook remains the most used social media platform, with over 2,800 million users worldwide (Statista, 2022). Please note that while writing this work, Facebook renamed itself to Meta describing the company being in charge for the Facebook, Instagram and WhatsApp services. The name change is intended to accompany the parent company’s move toward a changed business model (the ‘metaverse’) and a more immersive version of the social media experience, based on virtual reality technology (Isaac, 2021). For the present study, we will deal with the Facebook service.

Overall, social media applications have increased almost fivefold in their total user base in the last decade, passing from 970 million in 2010 to 4.48 billion users in July 2021 (Kemp, 2021). On average, during 2021, time spent on social media per day was 2 h and 27 min globally, for users aged 16–64 on any device (Kemp, 2021). The ever-growing popularity of social media can be traced back to its role in helping users fulfilling individual needs for self-expression and social belongingness (Casale and Fioravanti, 2018; Schivinski et al., 2020); still, its problematic use may have negative consequences on individuals’ mental health, especially among young users (Keles et al., 2020). At the moment there is a large debate if excessive or problematic use should be best understood as addictive behavior. In their analysis Brand et al. (2020) write for instance that the mechanisms underlying social networks use disorder (the term aligned with the officially recognized Gaming Disorder diagnosis in ICD-11) might be in parts comparable to that from Gaming Disorder, but the authors also mention that the “evidence with respect to functional impairment in daily life and findings from multi-methodological studies including clinical samples are arguably currently less convincing compared to pornography-use disorder and buying-shopping disorder”. In order to strive for unification of terminology in the study of Internet Use Disorders (Montag et al., 2021), we also use the term social networks use disorder (SNUD) from now on in this work, but mention that SNUD currently is not officially recognized as an official disorder and that we do not want to over-pathologize everyday life behavior (Billieux et al., 2015). Moreover, as we investigate subclinical samples, we speak in the present work of individual differences in tendencies toward SNUD.

An interesting aspect of social media is the fact that these platforms collect large amounts of data about their users. These data can be used for advertising purposes, but can also be used by academics to study digital footprints, which may consist of indices of user activity on social media (e.g., the number of posts, the number of Likes to online pages), audio and video data and have the advantage that they provide an “objective” representation of what users do online. Although this objective data is highly interesting, most existing social media studies dealing with SNUD rely on self-report of online activity, which may be prone to recall bias, social desirability and so forth. In particular, the study of the predictors and correlates of “Internet Use Disorders” and other related behavioral addictions could greatly benefit from the use of objective data (Montag et al., 2017; Ellis et al., 2018; Montag and Rumpf, 2021). Unfortunately, the use of these types of data is still uncommon, probably due to the technical complexity of the methodological tools needed to retrieve them. Moreover, at the moment Facebook’s APIs are closed for independent scientists, making it impossible for researchers to study actual behavior of its users (Montag et al., 2021). Therefore, it is not surprising that we only found a few studies investigating the association between user digital traces on social media and SNUD or excessive usage of the smartphone (i.e., Marino et al., 2017; Marengo et al., 2020; Peterka-Bonetta et al., 2021). The study by Marino et al. (2017) analyzed differences in objective digital footprints in problematic and non-problematic Facebook users. More in detail, they used a clustering technique to define groups of Facebook users based on their “Problematic Facebook Use” score. Findings indicated that problematic users had a higher number of friendships and increased wall activity compared to non-problematic users.

Beyond this study, Marengo et al. (2020) investigated the hypothesis that extraversion and neuroticism could be linked with tendencies toward SNUD because of their association with higher online posting activity, which they assessed by collecting digital traces of Facebook activity. Findings from this study highlighted that both the frequency of posting activity, and the number of Likes received, predicted higher tendencies toward SNUD among their users. Additionally, for both extraverts and neurotics, receiving more Likes because of increased posting activity predicted an increase in the risk for SNUD tendencies.

Language use in social media has also been effectively used in recent research to examine individual differences in online behavior (e.g., Settanni and Marengo, 2015; Reyes-Menendez et al., 2020). Specifically, language use has been shown to predict stable individual characteristics such as personality traits and intelligence (Azucar et al., 2018; Settanni et al., 2018), as well as more variable characteristics such as psychological well-being and stress (Settanni and Marengo, 2015; Marengo et al., 2021a,b), and risk behaviors (e.g., problematic alcohol use, Marengo et al., 2019). In general, the cited literature shows almost unanimously that models that include different types of digital traces as input tend to have increased predictive power when compared with models based on single digital traces.

Results from these studies are promising in suggesting that digital traces from social media could serve as a relevant source of information for the unobtrusive assessment of addictive usage tendencies toward social media platforms.

However, while the use of digital traces to make predictions on psychological variables is well established (Kosinski et al., 2013; Settanni et al., 2018; Marengo and Montag, 2020), studies exploring the feasibility of using digital traces to predict addictive tendencies toward social media are in large parts still missing. Also, against the background of discussions regarding ethical design of social media platforms, such research is of high importance (Montag and Hegelich, 2020).

With the present study, we aim to fill in this gap by studying if and how accurately addictive tendencies toward social media can be predicted by mining digital traces left on social media. To reach this goal, we examine the association between features extracted from digital traces of Facebook activity, and a validated measure of individual differences in SNUD (Bergen Social Media Addiction Scale, BSMAS). In particular, using machine learning methods we compare different set of features extracted from participants’ Facebook activity, including information about the posting activity, language use in Facebook posts, and expressed Likes, in terms of their overall contribution in predicting the scores of the BSMAS. Finally, we evaluate the feasibility of using the features extracted from digital traces of Facebook activity for the detection of users at risk for SNUD, as determined *via* using existing cut-offs.

## Materials and Methods

### Participants and Procedure

Participants were recruited among Italian Facebook users by disseminating online a specifically devised Facebook application allowing for both the administration of an online survey, as well as the collection of users’ digital footprints. In order to enroll in the study, participants had to be at least 18 years old, and provide access to their Facebook posts using the Facebook login. Informed consent was obtained by all participants using a specific online form. The research was approved by the universities’ institutional review board (n° 88,721, University of Turin). Participants were recruited *via* a snowball sampling approach, starting with a seed sample of 10 university students. Data collection took place from March to June 2018. Eventually, 3,000 participants accessed the app. 92.9% (*N* = 2,788) answered both at least one of the administered questionnaires in the online survey—such as a measure of personality or addictive social media use—and provided the researchers with access to their Facebook data, including posts and Likes data. Please note that part of the data presented here has been investigated already in other papers evaluating the association between personality, posting activity on Facebook, and social media addiction (Marengo et al., 2020), the association between language use on Facebook and quality of life (Marengo et al., 2021a), and the association between active Facebook use, self-esteem, and happiness (Marengo et al., 2021b).

For the purpose of the present study, we selected a subsample of 1,094 users for which both Facebook data and questionnaire response data on individual differences in the BSMAS were completely available. Please note that filling in the section of the online questionnaire including the BSMAS assessment was optional to participants; for this reason, a lower sample size was observed for this questionnaire (*N* = 1,094 out of a total of *N* = 2,788 participants, 39.2%). The sample analyzed here consisted mostly of Italian university students, of which a majority were females (female: *N* = 792; 72.4%; males: *N* = 302, 27.6%) and young adults (18–25 years old: *N* = 852, 77.9%; 26–35 years old: *N* = 189, 17.3%; and ≥36 years old: *N* = 53, 4.8%). As regards participants’ education level, *N* = 20 (1.8%) had at most a middle-school certification, *N* = 649 (59.3%) at most held a high school diploma, *N* = 319 (29.2%) at most held a bachelor’s degree, and *N* = 106 (9.7%) at most held at least a master’s degree.

Facebook Post and Likes data of these users were collected by submitting a request through the Facebook Graph application-programming interface (API). For the purpose of the present study, we examined posts and Likes data shared by users during the 12 months up until her/his access to the app and completion of the online questionnaire. Hence, Facebook data referring to the last 12 months of activity of the users were downloaded at the same time the users accessed and submitted their responses to the online questionnaire (for details on the administered questionnaire see section “Self-Report Inventory—Bergen Social Media Addiction Scale”).

### Measures

#### Features Extracted From Digital Traces of Facebook Activity

##### Posting Activity

We collected Facebook posting activity data by submitting requests through Facebook’s Graph API. The obtained data consisted of users’ posting activity data during the 12 months before the survey. Collected data included: total number of posts, number of textual posts, number of user’s comments to his/her own posts, publication time slots, number of profile updates, number of profile picture updates, number of posts in which the user tags other users, number of posts in which the user indicates to be somewhere, and number of posts in which the user tags other users and indicates to be somewhere. Additionally, along with each actual post posted by users, we obtained the number of Likes received by the post from other users. See Supplementary Table 1 for descriptive statistics of all features extracted from users’ recording of posting activity on Facebook.

##### Language Use

Automated text analysis was performed on the participants’ textual posts with the Linguistic Inquiry Word Count (LIWC) software. First, we aggregated all texts generated by participants in a single document per user, resulting in 1,094 documents. For each document, we computed a word count representing the total number of words posted by the user in the time window of 12 months of his/her Facebook activity before participating in the survey. Then, we scored the same documents using the LIWC dictionary (Pennebaker et al., 2007). For the purpose of this study, we employed an adapted version of the Italian version of the 2001 LIWC dictionary (Alparone et al., 2004). In its original version, the Italian LIWC includes 83 theory-based dictionary categories allowing for the scoring of documents based on the number of words reflecting for instance affective, social, cognitive, and perceptual processes, as well as use of function words such as pronouns, articles, conjunctions, verbs, and adverbs. For the present study, we extended the dictionary with six new custom dictionaries assessing use of emoticons and emoji. In order to include emoticons and emoji in the text analysis performed with LIWC, we used a two-step procedure. First, collected texts were pre-processed by recoding each distinct emoticon and emoji with a unique string id. This recoding step was necessary in order for emoticons and emoji to be detectable by the LIWC software. Then, we used the classification of emotional valence proposed by Vashisht and Thakur (2014) for emoticons, and Rodrigues et al. (2018) for emoji, to create six custom LIWC dictionary categories: emoticon use, emoticons showing positive and negative valence, emoji use, and emoji showing positive and negative valence. Overall, using this modified LIWC dictionary, we scored participants using 89 distinct dictionary categories (see Supplementary Table 2 for descriptive statistics regarding each of the extracted features).

##### Facebook Page Likes

We collected Facebook page Likes by submitting requests through Facebook’s Graph API. Features extracted included all expressed Likes to Facebook pages by the user since he/she joined Facebook, including a timestamp indicating when the user liked the page, and the category of the liked page. For the purpose of this study, because we are interested in factors affecting SNUD during the last year of activity, we limited our analyses to the last 12 months of Likes data. Additionally, because of the sparsity of the page Likes matrix (*N* = 46,827 pages for 1,094 users), we coded participants’ Likes on pages based on the page category information. For each Facebook page, the page category provides information about the type of content that users can expect to find on the page (e.g., Taylor Swift’s official Facebook page is categorized using the “Musician/band” category; Amazon’s page on Facebook is categorized as “Retail company”). Thus, for each user, we generated the count of Likes in each page category; eventually, this step resulted in the scoring of each participant based on 413 new features, each representing the total count of Likes expressed by the user to a specific page Likes category during the last 12 months before the survey. Additionally, over the same 12-month period, we compute the total number of Facebook page Likes expressed by the user, both in general and at different time slots.

#### Self-Report Inventory—Bergen Social Media Addiction Scale

We administered the Italian Bergen Social Media Addiction Scale (BSMAS, Andreassen et al., 2012; Monacis et al., 2017). Participants answered the questionnaire online. The BSMAS can be used to assess six components of social media addiction (in the present study used to assess SNUD): salience, tolerance, mood modification, relapse, withdrawal symptoms, and conflict. For the purpose of the present study, we asked participants to report about their symptoms during the last 12 months, using the following items: (1) “How often during the last year have you spent a lot of time thinking about social media or planned use of social media” (Salience); (2) “How often during the last year have you felt an urge to use social media more and more?” (Tolerance); (3) “How often during the last year have you used social media in order to forget about personal problems?” (Mood modification); (4) “How often during the last year have you tried to cut down on the use of social media without success?” (Relapse); (5) “How often during the last year have you become restless or troubled if you have been prohibited from using social media?” (Withdrawal symptoms); (6) “How often during the last year have you used social media so much that it has had a negative impact on your job/studies?” (Conflict). Participants rated each item on a 5-point scale (1 = very rarely, 5 = very often). Responses were summed to obtain a total score, resulting in a mean score of 14.18 (*SD* = 4.42, Observed Range = 6–28). The scale showed adequate reliability (Cronbach’s α = 0.76).

### Data Analysis

First, we used Spearman correlation coefficients to examine associations between the BSMAS score and features extracted from participants’ digital traces. We used Spearman correlations because of the lack of normality (i.e., positive skewness) of most of the features extracted from the digital traces. Because we perform a large number of correlations, as many as the extracted features (*N* = 526), we use Bonferroni correction to adjust *p*-values (i.e., we multiply the estimated *p*-values for the number of correlations performed, Jafari and Ansari-Pour, 2019).

Next, we examine if the features extracted from the digital traces left by users on Facebook can be used to predict their current BSMAS score (hence individual differences in tendencies toward SNUD). The predictive power of the extracted features was evaluated by implementing a series of predictive analyses based on the following set of features, analyzed separately or in combination with each other: posting activity, LIWC, and Likes, and a combination of all the features extracted from the digital traces. Finally, in order to check for the potential confounding effect of demographic variables (e.g., age, gender, and education level) in predicting the BSMAS, we ran two additional models and compared their results: a model including all features extracted from digital traces and demographic variables, and a final model including only the demographic variables.

The analyses were performed using a machine learning approach and involved the iterative splitting of the dataset into training and test sets including, respectively, 90% and 10% of observations. The training sets were used to train the predictive models, while the test sets were used to evaluate the accuracy of predictions on unseen observations. Because of the sparsity of our data, which includes as many as 526 features, we use this specific train/test split ratio instead of others (e.g., 70/30, or 80/20 splits) in order to retain as much data during the training of the models, while also retaining an adequate sample size in the test set (*n* ≈ 110). In order to improve generalizability of results to unseen observations, the splitting procedure and all analyses were repeated 10 times on different train/test splits, and then results were averaged across the 10 splits. Each of the 10 splits was obtained using the *partition()* function of the splitTools library for R (Mayer, 2020), which allowed us to generate samples for the training and test sets that were stratified according to the distribution of the BSMAS scores in the overall sample.

We performed prediction of the BSMAS continuous score using a stacking ensemble combing the Elastic Net (Zou and Hastie, 2005) and Random Forest (Breiman, 2001) algorithms, that is we used a “super learner” approach (Van der Laan et al., 2007). We chose to use a machine learning approach instead of standard parametric methods because using machine-learning methods can help to identify hidden interactions and non-linearity among features in predicting the outcome, and because they can help reduce the risk of overestimating the prediction performance (i.e., overfitting). Regarding the Elastic Net regression model, the alpha parameter was set at 1, resulting in a L1 parameter regularization (i.e., Least Absolute Shrinkage and Selection Operator, or LASSO regression; Tibshirani, 1996). The lambda parameter of the model was optimized by performing an automated search among a sequence of randomly generated 100 values. Lasso regression is a linear algorithm that relies on shrinkage of regression parameters to perform variable selection and regularization in order improve both model parsimony (i.e., models including fewer parameters) and prediction accuracy. In turn, the Random Forest algorithm is a non-linear algorithm essentially consisting in an ensemble of randomly generated decision trees, whose number is to be chosen by the researcher. For the purpose of this study, Random Forest regression was based on 1,000 trees, with unlimited depth of trees, and random selection of features. The stacking ensemble was implemented by combining the Elastic Net and Random Forest algorithms using a linear regression algorithm with no parameter regularization. The trained models were then evaluated on the test sets. Accuracy of predictions in the test sets was determined with the correlation between observed and predicted BSMAS scores, and both the mean absolute error (MAE) and root mean square error (RMSE) of predictions, computed as the mean of the absolute differences between observed and predicted scores. As noted above, results were averaged across the 10 test sets resulting from the 90/10 train/test splits. Prediction analyses were performed using the *Rweka* package in R (Hornik et al., 2020).

Finally, we selected the model generating the best performance in predicting the BSMAS and evaluated its performance in detecting those users’ falling in the category of clinically relevant SNUD according to cut-offs indicated by the literature. Here, we refer to two existing cut-offs: a 19-point cut off proposed by Bányai et al. (2017), and a stricter 24-point cut-off recently proposed by Luo et al. (2021). In order to determine the performance of the model, we compute the area under curve (AUC) values representing the association between participants’ predicted BSMAS (continuous) scores, and the binary classification of SNUD obtained by using the aforementioned cut-offs on the observed BSMAS score. That is, individuals scoring equal or above the cut-offs were labeled as at-risk users, while participating sitting below the cut-off were labeled as showing non-problematic SNUD. We performed this analysis separately on the *N* = 10 test sets, and then averaged the AUC values to obtain an overall indication of accuracy. Conceptually, in this context the AUC can be interpreted as the probability that a randomly selected case reporting an observed BSMAS score equal or above the suggested cut-off (i.e., an individual with probable social networks use disorder) would have a higher predicted score than randomly selected case with an observed BSMAS score below the cut-off. In following recommendations by Youngstrom (2014) for interpretation of AUC values based on questionnaire data, we deem AUC values in the 0.7–0.8 range as indicating an acceptable diagnostic performance going visibly beyond chance level.

## Results

### Associations Between Bergen Social Media Addiction Scale and Features Extracted From Facebook Digital Traces

Results of Spearman correlations analyses showed that, after adjusting *p*-values using a Bonferroni correction, 134 out of the 526 features extracted from participants’ digital traces on Facebook showed significant associations with the BSMAS. Please note that correlations between the BSMAS score and all features extracted from Facebook digital traces, including page Likes categories, are reported in the Supplementary Tables 1–3. Table 1 reports the emerging significant correlations for posting activity. Overall, the frequency of posting activity, as well the frequency of posts including a text, was generally positively correlated with the BSMAS scores, even though the frequency of texts shared during the night (i.e., between 0:00 and 5.59) showed a smaller effect size, and the frequency of posts during the same time slot was not significanly associated with the BSMAS score (see Supplementary Table 1). The number of Likes received by users’ posts was also positively associated with the BSMAS score.

**TABLE 1:** Spearman correlation between extracted features and the Bergen Social Media Addiction Scale (BSMAS) score.

Features	ρ	Adjusted. *P*
**Posting activity**		
Total number of posts	0.193	<0.001
Posts between 6:00 and 11:59	0.187	<0.001
Posts between 12:00 and 17.59	0.211	<0.001
Posts between 18:00 and 23.59	0.173	<0.001
Total number of textual posts	0.219	<0.001
Textual posts between 6:00 and 11:59	0.207	<0.001
Textual posts between 12:00 and 17:59	0.235	<0.001
Textual posts between 18:00 and 23:59	0.195	<0.001
Textual posts between 00:00 and 05:59	0.137	0.003
Total number of received Likes	0.204	<0.001
**Language**		
Word count	0.175	<0.001
*LIWC categories*		
Use of emoji in texts	0.220	<0.001
Positive Emoji	0.198	<0.001
Self	0.182	<0.001
I	0.180	<0.001
Certainty	0.174	<0.001
Article	0.172	<0.001
Time	0.172	<0.001
First person singular (verb)	0.169	<0.001
Negative emotions	0.165	<0.001
Present	0.165	<0.001
**Page Likes**		
Total Likes on Facebook pages	0.236	<0.001
Page Likes between 06:00 and 11:59	0.227	<0.001
Page Likes between 12:00 and 17:59	0.230	<0.001
Page Likes between 18:00 and 23:59	0.242	<0.001
Page Likes between 00:00 and 05:59	0.131	0.008
***Page Likes Categories***		
Artist	0.231	<0.001
Musician/band	0.220	<0.001
Public figure	0.220	<0.001
Entertainment website	0.203	<0.001
Media news company	0.203	<0.001
Community	0.189	<0.001
TV channel	0.172	<0.001
TV show	0.169	<0.001
Dance/night club	0.166	<0.001
Website	0.165	<0.001

*For LIWC and Likes categories, only categories reporting the top ten correlations are reported here.*

Concerning specifically the use of language, we found that the overall word count had a positive correlation with the BSMAS score. Additionally, 66 out of 89 LIWC dictionary categories showed positive correlations with the BSMAS. Table 1 reports the 10 LIWC categories reporting the highest correlation, while correlations between the BSMAS and all LIWC dictionary categories are reported in full in the Supplementary Table 2. Among those showing the strongest correlations with the BSMAS were use of emoji in texts (in particular those expressing positive sentiment), reference to self (e.g., *self* and *I* categories), words expressing negative emotions, use of words conveying certainty, words indicating references to time and dates, use of verbs conjugated in the first person singular, and present time-orientation.

Regarding Facebook Likes, the number of Likes expressed by the users’ on Facebook pages showed a positive correlation with the BSMAS score, although the number of Likes expressed over the night (0:00 and 5.59) showed a smaller effect size. Next, we saw that 52 out of 413 page Likes categories showed a significant association with the BSMAS score. Among the page categories showing the highest correlations with the BSMAS score, the top ten (see Table 1) were pages labeled by the creator as *artist*, *musician/band*, *public figure*, *entertainment website*, *media news company*, *community* (i.e., pages dedicated to a topic/experience/phenomenon that is owned collectively by the community connected to it), *TV channel*, *TV show*, *dance/night club*, and *Website*.

### Prediction of Tendencies Toward Social Networks Use Disorder Using Facebook Digital Traces

Table 2 presents the results of combining predictive analyses based on different set of features, and using a stacking ensemble of Random Forest algorithm and Lasso regression with linear regression as meta-classifier, for the prediction of the BSMAS. Overall, results showed that prediction was at best when all features extracted from digital traces were used (*r* = 0.32, MAE = 3.41, RMSE = 4.23), and when we combined features extracted from users’ posting activity, and Facebook Likes (*r* = 0.32, MAE = 3.42, RMSE = 4.24). In turn, the worst prediction performance was observed when the model included only language features extracted from users’ textual posts (*r* = 0.20, MAE = 3.54, RMSE = 4.38). Interestingly, when the model only included features extracted from Facebook Likes, the prediction performance was very close (*r* = 0.31, MAE = 3.42, RMSE = 4.26) to what was observed when all features were combined together.

**TABLE 2:** Average prediction performance of the BSMAS score basing on test sets.

Features in the model	*R*	MAE	RMSE
Posting activity	0.24	3.50	4.34
Language	0.20	3.54	4.38
Facebook Page Likes	0.31	3.42	4.26
Posting activity + language	0.25	3.50	4.33
Posting activity + Facebook page Likes	0.32	3.42	4.24
Language + Facebook page Likes	0.31	3.42	4.25
All features	0.32	3.41	4.23
All features + demographic variables	0.33	3.40	4.22
Demographic variables	0.08	3.60	4.45

*R, Correlation between observed and predicted BSMAS score; MAE, Mean Absolute Error; RMSE, Root Mean Square Error.*

Finally, regarding the overall contribution of demographic variables (i.e., age, gender, education level) to the prediction of the BSMAS, we saw that the inclusion of these variables along with the features extracted from digital traces only resulted in a minor increase in the predictive power of the model (*r* = 0.33, MAE = 3.40, RMSE = 4.22). In turn, when only demographic variables were included in the model, the predictive power was greatly diminished (*r* = 0.08, MAE = 3.60, RMSE = 4.45).

### Detection of Individuals With Probable Social Networks Use Disorder

Finally, we evaluated the performance of the best performing model, namely the model based on all the features extracted from the digital traces of Facebook activity, for the detection of individuals showing probably clinically relevant SNUD tendencies based on existing cut-off for the BSMAS. We saw that the average AUC values resulting from combining AUC values computed separately in 10 test sets were, respectively, 0.66 (Range: 0.57–0.72) for the 19-point cut-off, and 0.73 (Range: 0.49–0.93) for the 24-point cut-off. Hence, the detection of social media users at risk for SNUD tended to be more accurate when the higher cut off was used to distinguish between disordered and non-disordered social networks use.

## Discussion

The present study examined the associations between features extracted from digital traces of Facebook activity and a validated assessment of social networks use disorder, the BSMAS. In this work, we also compared the contribution of the features extracted from posting activity, language use, and Likes in predicting BSMAS scores. Finally, we evaluated the feasibility of using these digital footprint features to detect users at risk for SNUD (*via* defined cut-offs).

Consistent with the previous literature, overall frequency of posting activity and number of received Likes were found to be associated with BSMAS scores (Marino et al., 2017; Marengo et al., 2020). In terms of language use in social media texts, the BSMAS score was found to be associated with many aspects of language use. Among them, reference to self, use of words expressing both negative and positive emotions, use of emoji, and the use of verbs conjugated in the first person singular showed the strongest associations with the BSMAS scores. Combined these associations seem to indicate that users’ showing a pattern of online activity consisting of frequent online interpersonal exchanges, characterized by informal language (e.g., emoji), frequent emotional expressions, and relatively high positive social feedback from other users (e.g., received Likes), are more prone to show increased tendencies toward SNUD. These findings appear in line with those emerging from studies based on self-report data, and also those highlighting the role of social gratifications, need to belong and self-expression as important motives predicting social media use, and possibly addictive social media use (e.g., Seidman, 2013; Gao et al., 2017; Casale and Fioravanti, 2018).

An interesting aspect regarding frequency of posts, texts, and Likes expressed to Facebook pages is that the activity taking place during the night showed a weaker association with BSMAS scores when compared with records of user activity during the rest of the day. These observations seem to indicate that active social media use may be more addictive when it takes place when other users are more likely providing feedback to the user’s posts (e.g., commenting, Liking), as opposed to night hours, when many of other users do not respond to the user’s messages.

Likes expressed to Facebook pages by users also showed significant associations with their BSMAS score. The page categories for which Likes showed the strongest association with tendencies toward SNUD were related to the consumption of online and offline media and arts, including music, movies, television programs, and news. Please note that effect sizes are in the small area. Overall, these findings appear to be in line with studies highlighting the importance of entertainment and escapism motives driving social media use, and possibly even users’ tendencies toward SNUD (Gao et al., 2017; Leong et al., 2019).

As for the predictive analysis, findings from the present study indicate that the strongest predictive performance on new observations (hence the test data) can be achieved when all *digital footprint features* extracted from Facebook activity are included in a single model. At best, the (average) correlation between observed and predicted BSMAS scores was *r* = 0.32, which is in line with the literature investigating associations between digital traces of Facebook activity and many self-reported behavioral and psychological constructs (e.g., Settanni et al., 2018). Interestinlgy, including demographic variables along with the features extracted from digital traces resulted only in a minor increase in predictive performance (*r* = 0.33) of the model.

The present model achieved a similar performance even when only the features from Facebook page Likes were included. These results confirm the importance of Likes for the prediction of psychological constructs, extending previous studies by providing novel evidence regarding the role of Likes/digital footprints for the prediction of SNUD, which to our knowledge has not been studied before in this realm.

Finally, we saw that the model leveraging on all the features extracted from the digital traces of Facebook activity provided promising results in detecting users at risk for SNUD, especially when the more stringent 24-point cut-off was applied (Luo et al., 2021). This suggests the feasibility of implementing unobtrusive detection of individuals at risk by leveraging on users’ digital traces of online activity. Please see also a recent article discussing the potential of mobile sensing and digital phenotyping to combat Internet Use Disorders (Montag and Rumpf, 2021).

The present work is limited by its focus on Facebook. Recent work suggests that other platforms might be more “addictive” and results could differ there (Rozgonjuk et al., 2021). As a consequence, future studies should also study other social media platforms, although such an important research endeavor is limited by closed APIs of different platforms at the moment. Finally, larger samples, might help to test the robustness of our findings. Beyond this, the present study is limited by the investigation of a non-representative sample of Italian Facebook users (mostly university students), which limits the generalizability of the results to other social media platforms and other countries. Furthermore, the cross-sectional nature of the data used in this study prohibits to draw causal relationships. Although it is plausible that some design elements of social media trigger addictive tendencies toward the platforms (Montag et al., 2019), longitudinal studies are needed to establish such causality. Naturally, the large tech-companies have many relevant data to answer such questions, but usually these are not shared with independent academics or have been a disappointment (Hegelich, 2020). Finally, many inventories exist to assess individual differences in SNUD depending on different frameworks. As long as no consensus exists regarding the appropriate framework to be applied (addiction?), and no consensus exists regarding the symptoms and so forth, it will be difficult to compare studies in the field, which hinders scientific progress. The here applied BSMAS also asked for addictive tendencies toward social media/social networks in general, asking for addictive tendencies toward Facebook probably would have resulted in larger associations or accuracies.

## Conclusion

With the present study, we aimed to evaluate if and how accurately SNUD could be predicted by mining digital traces left on social media. For this purpose, first, we examined the association between features extracted from digital traces of Facebook activity, and a validated measure of SNUD. Then, we explored the overall predictive power of digital traces over SNUD using a machine learning approach. Overall, emerging results confirmed previous findings linking variables such as received Likes, wall-posting activities to individual differences in tendencies toward SNUD (e.g., Marino et al., 2017; Marengo et al., 2020). Results also highlighted the existence of a moderate predictive power of digital traces extracted from Facebook activity data in predicting SNUD, and confirmed the feasibility of this application to detect users at risk for SNUD.

## Data Availability Statement

The raw data supporting the conclusions of this article will be made available by the authors upon request. Requests to access the datasets should be directed to DM, davide.marengo@unito.it.

## Ethics Statement

The studies involving human participants were reviewed and approved by the IRB of University of Turin. Written informed consent for participation was not required for this study in accordance with the national legislation and the institutional requirements.

## Author Contributions

DM and MS designed the study and collected the data. DM and AM processed the data and ran statistical analysis. DM, AM, and MS wrote the first draft of the manuscript. CM worked over the complete manuscript and added several critical sections in the introduction and discussion. All authors contributed to the article and approved the submitted version.

## Conflict of Interest

CM mentions that he has received (to Ulm University and earlier University of Bonn) grants from agencies such as the German Research Foundation (DFG). CM has performed grant reviews for several agencies; has edited journal sections and articles; has given academic lectures in clinical or scientific venues or companies; and has generated books or book chapters for publishers of mental health texts. For some of these activities he received royalties, but never from gaming or social media companies. CM mentions that he is part of a discussion circle (Digitalität und Verantwortung: https://about.fb.com/de/news/h/gespraechskreis-digitalitaet-und-verantwortung/) debating ethical questions linked to social media, digitalization and society/democracy at Meta. In this context, he receives no salary for his activities. Finally, he mentions that he currently functions as independent scientist on the scientific advisory board of the Nymphenburg group (Munich, Germany) and Applied Cognition (Los Altos, CA, United States). This activity is financially compensated. The remaining authors declare that the research was conducted in the absence of any commercial or financial relationships that could be construed as a potential conflict of interest.

## Publisher’s Note

All claims expressed in this article are solely those of the authors and do not necessarily represent those of their affiliated organizations, or those of the publisher, the editors and the reviewers. Any product that may be evaluated in this article, or claim that may be made by its manufacturer, is not guaranteed or endorsed by the publisher.
