# Supplementary material for: Mining Digital Traces of Facebook Activity for the Prediction of Individual Differences in Tendencies Toward Social Networks Use Disorder: A Machine Learning Approach
Source: Front Psychol. 2022 Mar 8;13:830120. doi: 10.3389/fpsyg.2022.830120 (PMC8957912; doi:10.3389/fpsyg.2022.830120)
Supplement: Supplementary file 1 [file Data_Sheet_1.docx]

**Table S1. Descriptive statistics and Spearman correlation with the BSMAS for frequency of posting activity and received Likes**

|  | **M** | **SD** | **Range** | **ρ** | **Adj. p** |
| --- | --- | --- | --- | --- | --- |
| Posts |  |  |  |  |  |
| Total | 71.73 | 87.43 | 1-303 | .193 | <.001 |
| Posted between 06:00-11:59 | 21.42 | 28.24 | 0-154 | .187 | <.001 |
| Posted between 12:00-17:59 | 25.40 | 31.47 | 0-162 | .211 | <.001 |
| Posted between 18:00-23:59 | 22.39 | 29.3 | 0-178 | .173 | <.001 |
| Posted between 00:00-05:59 | 2.53 | 5.8 | 0-70 | .118 | .050 |
| Received Likes | 606.13 | 867.09 | 0-6301 | .204 | <.001 |
| Mean received Likes per post | 13.98 | 18.62 | 0-358 | .026 | 1.000 |
| Textual posts |  |  |  |  |  |
| Total | 37.82 | 52.99 | 0-288 | .219 | <.001 |
| Posted between 06:00-11:59 | 11.26 | 52.99 | 0-149 | .207 | <.001 |
| Posted between 12:00-17:59 | 13.59 | 19.58 | 0-123 | .235 | <.001 |
| Posted between 18:00-23:59 | 11,77 | 17.81 | 0-172 | .195 | <.001 |
| Posted between 00:00-05:59 | 1.19 | 2.83 | 0-34 | .137 | .003 |
| User’s comments on own posts | 3.41 | 7.47 | 0-73 | .096 | .758 |
| Profile updates | .52 | 2.29 | 0-44 | -.016 | 1.000 |
| Profile picture updates | .3 | 1.34 | 0-29 | -.016 | 1.000 |
| Other users’ tags | 1.14 | 4.74 | 0-68 | .046 | 1.000 |
| Posts including a place/location | .071 | 3.24 | 0-50 | .061 | 1.000 |
| Posts indicating the user is with other users | 1.07 | 4.15 | 0-49 | .031 | 1.000 |

**Table S2. Descriptive statistics and Spearman correlation with the BSMAS for features extracted from language use in Facebook posts**

|  | **M** | **SD** | **Range** | **ρ** | **Adj. p** |
| --- | --- | --- | --- | --- | --- |
| Word count | 709.64 | 1347.19 | 0-19470 | .175 | <.001 |
| LIWC: Total Pronoun | 41.21 | 9.55 | 0-1600 | .154 | <.001 |
| LIWC: I | 11.83 | 26.44 | 0-432 | .180 | <.001 |
| LIWC: We | 3.83 | 9.66 | 0-202 | .136 | .003 |
| LIWC: Self | 8.42 | 17.53 | 0-284 | .182 | <.001 |
| LIWC: You | 1.04 | 3.68 | 0-101 | .128 | .011 |
| LIWC: Other | 1.23 | 3.78 | 0-59 | .106 | .232 |
| LIWC: Negate | 14.65 | 33.25 | 0-611 | .149 | <.001 |
| LIWC: Assent | .47 | 1.31 | 0-18 | .099 | .575 |
| LIWC: Article | 69.01 | 14.91 | 0-2069 | .172 | <.001 |
| LIWC: Prepositions | 60.92 | 128.39 | 0-1837 | .141 | .001 |
| LIWC: Number | 1.15 | 2.78 | 0-35 | .142 | .001 |
| LIWC: Affective processes | 28.09 | 55.91 | 0-706 | .162 | <.001 |
| LIWC: Positive feelings | 16.42 | 32.37 | 0-372 | .156 | <.001 |
| LIWC: Positive emotions | 4.97 | 9.67 | 0-89 | .163 | <.001 |
| LIWC: Optimism and Energy | 3.81 | 7.70 | 0-78 | .128 | .012 |
| LIWC: Negative emotions | 10.92 | 23.09 | 0-318 | .165 | <.001 |
| LIWC: Anxiety | 1.39 | 3.10 | 0-39 | .120 | .039 |
| LIWC: Anger | 2.98 | 6.90 | 0-91 | .149 | <.001 |
| LIWC: Sadness | 4.39 | 9.30 | 0-131 | .159 | <.001 |
| LIWC: Cognitive processes | 34.40 | 73.91 | 0-1165 | .151 | <.001 |
| LIWC: Cause | 5.20 | 11.68 | 0-161 | .156 | <.001 |
| LIWC: Insight | 10.65 | 23.04 | 0-346 | .154 | <.001 |
| LIWC: Discrepancy | 12.77 | 28.15 | 0-504 | .138 | .002 |
| LIWC: Inhibition | 1.85 | 4.38 | 0-56 | .128 | .012 |
| LIWC: Tentativeness | 10.92 | 23.28 | 0-277 | .155 | <.001 |
| LIWC: Certainty | 7.31 | 14.50 | 0-184 | .174 | <.001 |
| LIWC: Sensory Processes | 7.59 | 17.07 | 0-305 | .146 | .001 |
| LIWC: See | 3.11 | 6.80 | 0-107 | .136 | .003 |
| LIWC: Hear | 2.85 | 7.57 | 0-152 | .124 | .019 |
| LIWC: Feel | 1.34 | 3.11 | 0-45 | .132 | .006 |
| LIWC: Social Processes | 22.53 | 49.44 | 0-916 | .130 | .009 |
| LIWC: Communication | 7.82 | 18.70 | 0-350 | .153 | <.001 |
| LIWC: References to others | 4.74 | 11.00 | 0-192 | .067 | 1.000 |
| LIWC: Friends | 1.10 | 2.65 | 0-33 | .090 | 1.000 |
| LIWC: Family | 1.68 | 4.81 | 0-70 | .088 | 1.000 |
| LIWC: Humans | 3.82 | 8.77 | 0-144 | .125 | .018 |
| LIWC: Time | 21.07 | 42.11 | 0-586 | .172 | <.001 |
| LIWC: Past | 7.60 | 17.43 | 0-257 | .148 | <.001 |
| LIWC: Present | 45.65 | 96.72 | 0-1602 | .165 | <.001 |
| LIWC: Future | 1.89 | 4.07 | 0-53 | .153 | <.001 |
| LIWC: Space | 5.95 | 12.09 | 0-180 | .160 | <.001 |
| LIWC: Up | 1.59 | 3.76 | 0-48 | .133 | .006 |
| LIWC: Down | .36 | .99 | 0-13 | .113 | .095 |
| LIWC: Inclusion | 9.16 | 19.57 | 0-309 | .143 | .001 |
| LIWC: Exclusion | 27.30 | 62.78 | 0-1032 | .154 | <.001 |
| LIWC: Motion | 7.70 | 16.76 | 0-261 | .146 | .001 |
| LIWC: Occupation | 6.04 | 13.22 | 0-144 | .145 | .001 |
| LIWC: School | 1.94 | 4.80 | 0-66 | .128 | .011 |
| LIWC: Job | 1.01 | 2.86 | 0-31 | .119 | .044 |
| LIWC: Achievement | 3.70 | 7.86 | 0-83 | .136 | .003 |
| LIWC: Leisure Activity | 3.77 | 8.46 | 0-131 | .146 | .001 |
| LIWC: Home | 2.29 | 5.01 | 0-72 | .136 | .003 |
| LIWC: Sports | .34 | 1.37 | 0-27 | .049 | 1.000 |
| LIWC: TV | .56 | 2.95 | 0-72 | .136 | .003 |
| LIWC: Music | .53 | 1.38 | 0-14 | .139 | .002 |
| LIWC: Money & Financial Issues | 1.30 | 3.46 | 0-51 | .117 | .053 |
| LIWC: Metaphysical Issues | 2.47 | 5.38 | 0-83 | .154 | <.001 |
| LIWC: Religion | 1.66 | 3.62 | 0-48 | .146 | .001 |
| LIWC: Death & dying | .83 | 2.26 | 0-34 | .148 | .001 |
| LIWC: Physical Functions | 7.22 | 15.00 | 0-229 | .151 | <.001 |
| LIWC: Body states, symptoms | 4.63 | 9.98 | 0-159 | .128 | .011 |
| LIWC: Sex and sexuality | 1.38 | 3.47 | 0-42 | .140 | .002 |
| LIWC: Eating, drinking, dieting | .90 | 2.47 | 0-36 | .098 | .618 |
| LIWC: Sleeping, dreaming | .89 | 2.15 | 0-28 | .117 | .052 |
| LIWC: Grooming | .30 | .90 | 0-8 | .071 | 1.000 |
| LIWC: Swear words | .61 | 1.95 | 0-28 | .080 | 1.000 |
| LIWC: Non fluency words | .80 | 2.25 | 0-24 | .116 | .066 |
| LIWC: Filler words | 1.58 | 3.82 | 0-46 | .108 | .172 |
| LIWC: You (plural) | .02 | .16 | 0-2 | .024 | 1.000 |
| LIWC: He/Her | .35 | 1.29 | 0-23 | .061 | 1.000 |
| LIWC: Them | .02 | .18 | 0-4 | .042 | 1.000 |
| LIWC: Conditional verb | 4.87 | 11.60 | 0-161 | .140 | .002 |
| LIWC: Transitive verb | 3.58 | 7.75 | 0-101 | .142 | .001 |
| LIWC: Past participle | 9.56 | 21.10 | 0-288 | .112 | .109 |
| LIWC: Gerund | .68 | 1.73 | 0-19 | .083 | 1.000 |
| LIWC: To be | 4.72 | 1.12 | 0-163 | .125 | .018 |
| LIWC: To have | 6.11 | 15.36 | 0-290 | .132 | .006 |
| LIWC: First person singular (verb) | 13.13 | 31.03 | 0-612 | .169 | <.001 |
| LIWC: Second person singular (verb) | 4.42 | 8.92 | 0-91 | .134 | .005 |
| LIWC: Third person singular (verb) | 12.11 | 27.66 | 0-421 | .161 | <.001 |
| LIWC: First person plural (verb) | 2.48 | 5.29 | 0-79 | .130 | .009 |
| LIWC: Second person plural (verb) | 1.91 | 4.66 | 0-57 | .100 | .492 |
| LIWC: Third person plural (verb) | 3.96 | 1.38 | 0-194 | .132 | .006 |
| LIWC: Use of emoticons in texts | 1.97 | 7.13 | 0-144 | .070 | 1.000 |
| LIWC: Positive Emoticon | 1.87 | 6.96 | 0-144 | .072 | 1.000 |
| LIWC: Negative Emoticon | .09 | .48 | 0-7 | .040 | 1.000 |
| LIWC: Use of emoji in texts | 33.89 | 7.87 | 0-814 | .220 | <.001 |
| LIWC: Positive Emoji | 16.33 | 39.25 | 0-496 | .198 | <.001 |
| LIWC: Negative Emoji | 1.65 | 4.747 | 0-53 | .140 | .002 |

**Table S3. Descriptive statistics and Spearman correlation with the BSMAS for features extracted from Facebook Likes**

|  | **M** | **SD** | **Range** | **ρ** | **Adj. p** |
| --- | --- | --- | --- | --- | --- |
| Total Likes on Facebook pages | 74.37 | 117.03 | 1-1842 | .236 | <.001 |
| Page Likes between 06:00-11:59 | 21.11 | 34.71 | 0-508 | .227 | <.001 |
| Page Likes between 12:00-17:59 | 25.9 | 4.23 | 0-501 | .230 | <.001 |
| Page Likes between 18:00-23:59 | 23.49 | 4.93 | 0-763 | .242 | <.001 |
| Page Likes between 00:00-05:59 | 3.87 | 7.94 | 0-91 | .131 | .008 |
| likes_academiccamp | .01 | .10 | 0-1 | .028 | 1.000 |
| likes_accessories | .04 | .21 | 0-2 | .076 | 1.000 |
| likes_actor | .25 | 2.03 | 0-63 | .094 | .936 |
| likes_advertisingagency | .09 | .60 | 0-9 | .123 | .024 |
| likes_advertisingmarketing | .05 | .30 | 0-4 | .065 | 1.000 |
| likes_aerospacecompany | .03 | .33 | 0-8 | .071 | 1.000 |
| likes_agriculturalservice | .02 | .13 | 0-2 | .010 | 1.000 |
| likes_agriculture | .07 | .30 | 0-3 | .051 | 1.000 |
| likes_agriculturecompany | .05 | .27 | 0-3 | .007 | 1.000 |
| likes_airlinecompany | .05 | .29 | 0-5 | .040 | 1.000 |
| likes_album | .09 | .34 | 0-4 | .091 | 1.000 |
| likes_amateursportsteam | .14 | .54 | 0-8 | .031 | 1.000 |
| likes_americanrestaurant | .02 | .16 | 0-2 | .049 | 1.000 |
| likes_amusementthemepark | .03 | .17 | 0-2 | -.003 | 1.000 |
| likes_animal | .02 | .13 | 0-1 | .055 | 1.000 |
| likes_animalshelter | .01 | .11 | 0-2 | .016 | 1.000 |
| likes_apparelclothing | .04 | .22 | 0-3 | .030 | 1.000 |
| likes_appliances | .01 | .11 | 0-1 | .021 | 1.000 |
| likes_apppage | .22 | .65 | 0-7 | .119 | .039 |
| likes_architecturaldesigner | .01 | .13 | 0-2 | .009 | 1.000 |
| likes_art | .52 | 2.71 | 0-77 | .116 | .061 |
| likes_artgallery | .10 | .44 | 0-4 | .110 | .144 |
| likes_artist | 4.15 | 11.19 | 0-315 | .231 | <.001 |
| likes_artmuseum | .09 | .49 | 0-7 | .091 | 1.000 |
| likes_artschool | .02 | .16 | 0-2 | .023 | 1.000 |
| likes_artsentertainment | .89 | 1.87 | 0-25 | .144 | .001 |
| likes_artshumanitieswebsite | .32 | .90 | 0-10 | .119 | .044 |
| likes_athlete | .53 | 1.79 | 0-24 | .097 | .696 |
| likes_author | .44 | 1.64 | 0-39 | .161 | <.001 |
| likes_automotiveaircraftboa | .02 | .18 | 0-3 | .055 | 1.000 |
| likes_automotivebodyshop | .01 | .11 | 0-2 | -.015 | 1.000 |
| likes_babygoodskidsgoods | .05 | .28 | 0-5 | .068 | 1.000 |
| likes_bagsluggage | .07 | .37 | 0-4 | .031 | 1.000 |
| likes_bakery | .03 | .26 | 0-6 | -.029 | 1.000 |
| likes_band | .17 | .63 | 0-8 | .085 | 1.000 |
| likes_bar | .24 | .69 | 0-9 | .052 | 1.000 |
| likes_barbershop | .02 | .14 | 0-1 | .054 | 1.000 |
| likes_bargrill | .02 | .16 | 0-2 | .041 | 1.000 |
| likes_beach | .04 | .23 | 0-3 | -.010 | 1.000 |
| likes_beachresort | .01 | .11 | 0-2 | .012 | 1.000 |
| likes_beautycosmeticpersona | .11 | .41 | 0-5 | .009 | 1.000 |
| likes_beautysalon | .04 | .25 | 0-5 | .039 | 1.000 |
| likes_beautysupplystore | .01 | .10 | 0-1 | .036 | 1.000 |
| likes_bedandbreakfast | .06 | .28 | 0-4 | .080 | 1.000 |
| likes_beerbar | .02 | .17 | 0-3 | .108 | .193 |
| likes_bicycleshop | .01 | .11 | 0-2 | .009 | 1.000 |
| likes_bikerental | .02 | .12 | 0-1 | .019 | 1.000 |
| likes_biotechnologycompany | .03 | .32 | 0-8 | .019 | 1.000 |
| likes_blogger | .63 | 1.26 | 0-19 | .148 | <.001 |
| likes_boardgame | .03 | .33 | 0-7 | .066 | 1.000 |
| likes_book | .30 | 1.04 | 0-16 | .135 | .004 |
| likes_bookseries | .06 | .33 | 0-7 | .017 | 1.000 |
| likes_bookstore | .26 | .82 | 0-8 | .075 | 1.000 |
| likes_brand | .16 | .47 | 0-4 | .156 | <.001 |
| likes_brewery | .05 | .35 | 0-5 | .046 | 1.000 |
| likes_bridalshop | .03 | .22 | 0-4 | .029 | 1.000 |
| likes_broadcastingmediaprodu | .08 | .30 | 0-3 | .069 | 1.000 |
| likes_burgerrestaurant | .07 | .34 | 0-4 | .049 | 1.000 |
| likes_businesseconomywebsite | .05 | .23 | 0-3 | .109 | .165 |
| likes_businessservice | .13 | .46 | 0-4 | .080 | 1.000 |
| likes_busline | .02 | .15 | 0-2 | .055 | 1.000 |
| likes_butchershop | .01 | .11 | 0-1 | -.015 | 1.000 |
| likes_cafe | .07 | .32 | 0-4 | .055 | 1.000 |
| likes_cameraphoto | .06 | .29 | 0-4 | .087 | 1.000 |
| likes_campusbuilding | .01 | .12 | 0-1 | .052 | 1.000 |
| likes_candystore | .02 | .14 | 0-2 | .039 | 1.000 |
| likes_cardealership | .02 | .22 | 0-4 | .082 | 1.000 |
| likes_cargofreightcompany | .02 | .16 | 0-3 | .052 | 1.000 |
| likes_carrental | .01 | .10 | 0-1 | .019 | 1.000 |
| likes_cars | .07 | .48 | 0-8 | .045 | 1.000 |
| likes_cause | .13 | .50 | 0-6 | .145 | .001 |
| likes_charityorganization | .03 | .20 | 0-2 | .069 | 1.000 |
| likes_chef | .12 | .40 | 0-4 | .100 | .484 |
| likes_chemicalcompany | .02 | .17 | 0-2 | .056 | 1.000 |
| likes_chocolateshop | .01 | .12 | 0-2 | .046 | 1.000 |
| likes_churchofjesuschristof | .01 | .11 | 0-1 | .088 | 1.000 |
| likes_city | .11 | .38 | 0-4 | .099 | .573 |
| likes_cityhall | .01 | .11 | 0-1 | -.037 | 1.000 |
| likes_clothingbrand | .93 | 2.20 | 0-35 | .158 | <.001 |
| likes_clothingstore | .23 | .93 | 0-22 | .112 | .106 |
| likes_coach | .13 | .52 | 0-8 | .064 | 1.000 |
| likes_cocktailbar | .07 | .33 | 0-4 | .045 | 1.000 |
| likes_coffeeshop | .10 | .42 | 0-5 | .085 | 1.000 |
| likes_collectiblesstore | .01 | .12 | 0-2 | .047 | 1.000 |
| likes_collegeuniversity | .79 | 1.76 | 0-24 | .146 | .001 |
| likes_comedian | .71 | 1.75 | 0-23 | .153 | <.001 |
| likes_comicbookstore | .03 | .19 | 0-3 | .033 | 1.000 |
| likes_community | 7.64 | 13.55 | 0-180 | .189 | <.001 |
| likes_communitycenter | .02 | .15 | 0-2 | .067 | 1.000 |
| likes_communitycollege | .19 | .50 | 0-5 | .078 | 1.000 |
| likes_communityorganization | .31 | .83 | 0-9 | .076 | 1.000 |
| likes_communityservice | .06 | .28 | 0-4 | .061 | 1.000 |
| likes_company | .48 | 1.19 | 0-10 | .123 | .024 |
| likes_computercompany | .07 | .32 | 0-4 | .048 | 1.000 |
| likes_computersinternetwebsi | .04 | .23 | 0-4 | .048 | 1.000 |
| likes_computertrainingschool | .01 | .10 | 0-1 | .005 | 1.000 |
| likes_concerttour | .32 | .89 | 0-8 | .123 | .024 |
| likes_constructioncompany | .01 | .14 | 0-3 | .025 | 1.000 |
| likes_consultingagency | .21 | .81 | 0-14 | .104 | .289 |
| likes_contemporaryartmuseum | .03 | .22 | 0-4 | .047 | 1.000 |
| likes_cookingschool | .03 | .17 | 0-2 | .022 | 1.000 |
| likes_cosmeticsstore | .06 | .31 | 0-5 | .023 | 1.000 |
| likes_counselor | .02 | .16 | 0-2 | .080 | 1.000 |
| likes_country | .01 | .11 | 0-2 | .018 | 1.000 |
| likes_culturalcenter | .10 | .40 | 0-6 | .094 | .938 |
| likes_cupcakeshop | .07 | .30 | 0-2 | .041 | 1.000 |
| likes_dancenightclub | .40 | 1.17 | 0-17 | .166 | <.001 |
| likes_dancer | .10 | .40 | 0-4 | .052 | 1.000 |
| likes_dancestudio | .03 | .20 | 0-2 | .062 | 1.000 |
| likes_daycare | .01 | .14 | 0-3 | -.014 | 1.000 |
| likes_deli | .04 | .23 | 0-4 | .068 | 1.000 |
| likes_designfashion | .05 | .24 | 0-4 | .062 | 1.000 |
| likes_dessertshop | .02 | .19 | 0-4 | .018 | 1.000 |
| likes_diner | .05 | .24 | 0-3 | .026 | 1.000 |
| likes_divebar | .05 | .27 | 0-4 | .032 | 1.000 |
| likes_dj | .05 | .35 | 0-8 | .029 | 1.000 |
| likes_doctor | .06 | .29 | 0-4 | .048 | 1.000 |
| likes_dogtrainer | .03 | .17 | 0-2 | -.016 | 1.000 |
| likes_ecommercewebsite | .08 | .34 | 0-4 | .065 | 1.000 |
| likes_education | .68 | 1.74 | 0-22 | .094 | .931 |
| likes_educationalconsultant | .01 | .13 | 0-3 | .027 | 1.000 |
| likes_educationalresearchcente | .03 | .20 | 0-3 | -.034 | 1.000 |
| likes_educationcompany | .02 | .12 | 0-1 | .011 | 1.000 |
| likes_educationwebsite | .19 | .77 | 0-18 | .042 | 1.000 |
| likes_electronics | .03 | .20 | 0-2 | .065 | 1.000 |
| likes_electronicscompany | .05 | .21 | 0-1 | .050 | 1.000 |
| likes_electronicsstore | .01 | .14 | 0-3 | .025 | 1.000 |
| likes_elementaryschool | .02 | .14 | 0-2 | -.034 | 1.000 |
| likes_energycompany | .02 | .17 | 0-3 | .057 | 1.000 |
| likes_engineeringservice | .01 | .12 | 0-3 | .019 | 1.000 |
| likes_entertainmentwebsite | .92 | 2.47 | 0-40 | .203 | <.001 |
| likes_entrepreneur | .14 | .46 | 0-7 | .121 | .033 |
| likes_environmentalconservation | .08 | .36 | 0-4 | .095 | .901 |
| likes_escapegameroom | .03 | .20 | 0-3 | .052 | 1.000 |
| likes_event | .38 | .90 | 0-8 | .129 | .010 |
| likes_eventplanner | .38 | .96 | 0-15 | .144 | .001 |
| likes_exchangeprogram | .02 | .14 | 0-2 | .008 | 1.000 |
| likes_fashion | .02 | .14 | 0-1 | .045 | 1.000 |
| likes_fashioncompany | .01 | .11 | 0-1 | .029 | 1.000 |
| likes_fashiondesigner | .02 | .15 | 0-2 | .062 | 1.000 |
| likes_fashionmodel | .04 | .32 | 0-7 | .071 | 1.000 |
| likes_fastfoodrestaurant | .14 | .51 | 0-6 | .094 | .937 |
| likes_festival | .29 | .84 | 0-8 | .111 | .125 |
| likes_fictionalcharacter | .54 | 1.94 | 0-43 | .146 | .001 |
| likes_filmdirector | .06 | .32 | 0-7 | .109 | .156 |
| likes_financecompany | .02 | .14 | 0-2 | .051 | 1.000 |
| likes_financialservice | .02 | .20 | 0-4 | .093 | 1.000 |
| likes_fleamarket | .04 | .21 | 0-2 | .020 | 1.000 |
| likes_florist | .01 | .12 | 0-2 | .016 | 1.000 |
| likes_foodbeverage | .12 | .40 | 0-4 | .084 | 1.000 |
| likes_foodbeveragecompany | .37 | 1.40 | 0-29 | .120 | .039 |
| likes_fooddeliveryservice | .01 | .11 | 0-1 | .019 | 1.000 |
| likes_foodservicedistributor | .03 | .17 | 0-1 | .083 | 1.000 |
| likes_footwearstore | .06 | .29 | 0-3 | .090 | 1.000 |
| likes_funeralservicecemetery | .01 | .13 | 0-2 | .015 | 1.000 |
| likes_furniture | .05 | .27 | 0-4 | .038 | 1.000 |
| likes_furniturestore | .02 | .18 | 0-4 | .016 | 1.000 |
| likes_gamestoys | .16 | .82 | 0-20 | .025 | 1.000 |
| likes_gastropub | .07 | .29 | 0-4 | .022 | 1.000 |
| likes_gelatoshop | .01 | .13 | 0-2 | .073 | 1.000 |
| likes_giftshop | .05 | .24 | 0-3 | .039 | 1.000 |
| likes_governmentofficial | .03 | .27 | 0-6 | .085 | 1.000 |
| likes_governmentorganization | .47 | 1.36 | 0-15 | .104 | .286 |
| likes_graphicdesigner | .08 | .58 | 0-15 | .106 | .229 |
| likes_grocerystore | .07 | .30 | 0-4 | .083 | 1.000 |
| likes_gymphysicalfitnesscente | .15 | .45 | 0-4 | .047 | 1.000 |
| likes_hairsalon | .12 | .46 | 0-6 | .080 | 1.000 |
| likes_halfwayhouse | .01 | .10 | 0-1 | .046 | 1.000 |
| likes_healthbeauty | .70 | 1.67 | 0-22 | .145 | .001 |
| likes_healthfoodstore | .01 | .11 | 0-2 | -.006 | 1.000 |
| likes_healthspa | .05 | .25 | 0-3 | .037 | 1.000 |
| likes_healthwellnesswebsite | .19 | .80 | 0-18 | .097 | .725 |
| likes_highschool | .03 | .17 | 0-2 | .039 | 1.000 |
| likes_historymuseum | .11 | .39 | 0-4 | .082 | 1.000 |
| likes_home | .04 | .21 | 0-2 | .073 | 1.000 |
| likes_homedecor | .07 | .34 | 0-5 | .071 | 1.000 |
| likes_homegardenwebsite | .02 | .17 | 0-4 | -.031 | 1.000 |
| likes_homeimprovement | .03 | .20 | 0-4 | .001 | 1.000 |
| likes_hospital | .02 | .20 | 0-3 | .049 | 1.000 |
| likes_hostel | .02 | .16 | 0-2 | .024 | 1.000 |
| likes_hotel | .11 | .39 | 0-4 | .053 | 1.000 |
| likes_hotellodging | .02 | .16 | 0-2 | .050 | 1.000 |
| likes_hotelresort | .05 | .28 | 0-4 | .024 | 1.000 |
| likes_householdsupplies | .01 | .11 | 0-1 | .001 | 1.000 |
| likes_icecreamshop | .07 | .30 | 0-3 | .031 | 1.000 |
| likes_industrialcompany | .02 | .16 | 0-2 | .075 | 1.000 |
| likes_inn | .01 | .10 | 0-1 | .026 | 1.000 |
| likes_insuranceagent | .01 | .10 | 0-1 | .002 | 1.000 |
| likes_insurancecompany | .02 | .14 | 0-2 | .045 | 1.000 |
| likes_interest | .17 | 1.14 | 0-35 | .137 | .003 |
| likes_interiordesignstudio | .03 | .19 | 0-3 | .033 | 1.000 |
| likes_internetcompany | .08 | .32 | 0-3 | .083 | 1.000 |
| likes_italianrestaurant | .23 | .77 | 0-11 | .070 | 1.000 |
| likes_jazzbluesclub | .02 | .15 | 0-1 | .031 | 1.000 |
| likes_jewelrywatches | .19 | .69 | 0-12 | .042 | 1.000 |
| likes_jewelrywatchesstore | .03 | .20 | 0-2 | .024 | 1.000 |
| likes_journalist | .16 | .58 | 0-11 | .118 | .050 |
| likes_justforfun | .74 | 2.45 | 0-51 | .101 | .442 |
| likes_kitchencooking | .12 | .56 | 0-14 | .090 | 1.000 |
| likes_laborunion | .01 | .12 | 0-2 | .003 | 1.000 |
| likes_landmarkhistoricalplac | .14 | .47 | 0-5 | .083 | 1.000 |
| likes_languageschool | .05 | .26 | 0-3 | .005 | 1.000 |
| likes_lawyerlawfirm | .04 | .40 | 0-11 | .076 | 1.000 |
| likes_library | .14 | .54 | 0-9 | .123 | .022 |
| likes_lifestyleservice | .01 | .10 | 0-1 | -.013 | 1.000 |
| likes_literaryarts | .12 | .50 | 0-8 | .095 | .864 |
| likes_livemusicvenue | .06 | .30 | 0-4 | .035 | 1.000 |
| likes_localbusiness | .46 | 1.33 | 0-31 | .046 | 1.000 |
| likes_localservice | .05 | .25 | 0-3 | .048 | 1.000 |
| likes_localtravelwebsite | .33 | .83 | 0-8 | .120 | .035 |
| likes_lounge | .05 | .28 | 0-6 | .094 | 1.000 |
| likes_magazine | 1.02 | 2.54 | 0-32 | .153 | <.001 |
| likes_makeupartist | .01 | .12 | 0-1 | .034 | 1.000 |
| likes_marketingagency | .02 | .16 | 0-2 | .037 | 1.000 |
| likes_martialartsschool | .03 | .31 | 0-8 | .015 | 1.000 |
| likes_massageservice | .02 | .15 | 0-2 | .022 | 1.000 |
| likes_media | .20 | .63 | 0-7 | .099 | .578 |
| likes_mediaagency | .03 | .17 | 0-2 | .088 | 1.000 |
| likes_medianewscompany | 2.34 | 4.48 | 0-70 | .203 | <.001 |
| likes_medicalcenter | .02 | .14 | 0-2 | -.001 | 1.000 |
| likes_medicalcompany | .09 | .43 | 0-5 | .044 | 1.000 |
| likes_medicalhealth | .24 | .73 | 0-9 | .066 | 1.000 |
| likes_medicalresearchcenter | .02 | .19 | 0-5 | .066 | 1.000 |
| likes_meetingroom | .03 | .17 | 0-1 | .037 | 1.000 |
| likes_mentalhealthservice | .01 | .13 | 0-2 | -.055 | 1.000 |
| likes_mobilephoneshop | .01 | .12 | 0-2 | .063 | 1.000 |
| likes_modelingagency | .03 | .26 | 0-6 | .073 | 1.000 |
| likes_modernartmuseum | .03 | .23 | 0-3 | .042 | 1.000 |
| likes_monarch | .02 | .18 | 0-3 | .046 | 1.000 |
| likes_moroccanrestaurant | .01 | .10 | 0-1 | -.012 | 1.000 |
| likes_motivationalspeaker | .04 | .27 | 0-5 | .105 | .252 |
| likes_motorvehiclecompany | .04 | .24 | 0-3 | .035 | 1.000 |
| likes_mountain | .03 | .20 | 0-3 | .057 | 1.000 |
| likes_movie | 1.00 | 7.74 | 0-246 | .158 | <.001 |
| likes_moviecharacter | .07 | .40 | 0-8 | .086 | 1.000 |
| likes_movietelevisionstudio | .18 | .75 | 0-19 | .107 | .196 |
| likes_movietheater | .46 | 2.03 | 0-49 | .084 | 1.000 |
| likes_museum | .07 | .37 | 0-6 | .055 | 1.000 |
| likes_museumartgallery | .01 | .12 | 0-2 | .013 | 1.000 |
| likes_music | .04 | .22 | 0-3 | .097 | .725 |
| likes_musicalinstrument | .03 | .24 | 0-6 | .033 | 1.000 |
| likes_musicaward | .03 | .22 | 0-3 | .082 | 1.000 |
| likes_musicchart | .02 | .14 | 0-2 | .061 | 1.000 |
| likes_musician | .24 | .72 | 0-10 | .161 | <.001 |
| likes_musicianband | 2.70 | 6.13 | 0-92 | .220 | <.001 |
| likes_musiclessonsinstructio | .01 | .11 | 0-2 | -.003 | 1.000 |
| likes_musicproductionstudio | .02 | .13 | 0-2 | .087 | 1.000 |
| likes_musicvideo | .03 | .21 | 0-3 | .069 | 1.000 |
| likes_nailsalon | .01 | .10 | 0-1 | -.001 | 1.000 |
| likes_newsmediawebsite | 1.20 | 2.13 | 0-32 | .149 | <.001 |
| likes_newspaper | .17 | .52 | 0-5 | .066 | 1.000 |
| likes_newspersonality | .03 | .18 | 0-2 | .095 | .835 |
| likes_nongovernmentalorganizat | .19 | .66 | 0-9 | .116 | .062 |
| likes_nonprofitorganization | 2.55 | 5.02 | 0-61 | .116 | .065 |
| likes_nutritionist | .02 | .17 | 0-2 | .039 | 1.000 |
| likes_officesupplies | .02 | .17 | 0-2 | -.020 | 1.000 |
| likes_operahouse | .02 | .19 | 0-5 | .012 | 1.000 |
| likes_organization | .91 | 1.93 | 0-22 | .134 | .005 |
| likes_other | .05 | .25 | 0-3 | .024 | 1.000 |
| likes_outdoorsportinggoodsc | .04 | .26 | 0-4 | .058 | 1.000 |
| likes_outletstore | .03 | .17 | 0-2 | .082 | 1.000 |
| likes_park | .04 | .27 | 0-5 | .009 | 1.000 |
| likes_partyentertainmentservic | .01 | .11 | 0-1 | .024 | 1.000 |
| likes_performanceart | .18 | .68 | 0-12 | .106 | .246 |
| likes_performancearttheatre | .23 | 2.43 | 0-78 | .084 | 1.000 |
| likes_performanceeventvenue | .28 | .75 | 0-8 | .159 | <.001 |
| likes_performingarts | .52 | 1.16 | 0-10 | .122 | .027 |
| likes_performingartsschool | .02 | .14 | 0-2 | .010 | 1.000 |
| likes_personalblog | 1.66 | 4.03 | 0-81 | .162 | <.001 |
| likes_personaltrainer | .03 | .23 | 0-5 | .064 | 1.000 |
| likes_personalwebsite | .04 | .28 | 0-6 | .062 | 1.000 |
| likes_pet | .23 | .67 | 0-12 | .156 | <.001 |
| likes_petbreeder | .04 | .57 | 0-17 | .052 | 1.000 |
| likes_petservice | .06 | .43 | 0-8 | .080 | 1.000 |
| likes_petsupplies | .03 | .24 | 0-4 | -.007 | 1.000 |
| likes_pharmaceuticals | .02 | .22 | 0-6 | .014 | 1.000 |
| likes_pharmacydrugstore | .04 | .24 | 0-3 | .012 | 1.000 |
| likes_phonetablet | .01 | .10 | 0-1 | -.002 | 1.000 |
| likes_photographer | .57 | 1.89 | 0-40 | .144 | .001 |
| likes_photographyvideography | .02 | .18 | 0-2 | .028 | 1.000 |
| likes_physicaltherapist | .02 | .16 | 0-2 | .016 | 1.000 |
| likes_piedmontrestaurant | .01 | .13 | 0-2 | .033 | 1.000 |
| likes_pizzaplace | .24 | .82 | 0-14 | .067 | 1.000 |
| likes_plasticsurgeon | .01 | .19 | 0-6 | .014 | 1.000 |
| likes_podcast | .04 | .22 | 0-4 | .056 | 1.000 |
| likes_politicalcandidate | .06 | .28 | 0-4 | .041 | 1.000 |
| likes_politicalorganization | .48 | 1.66 | 0-22 | .102 | .379 |
| likes_politicalparty | .13 | .60 | 0-9 | .063 | 1.000 |
| likes_politician | .42 | 1.50 | 0-21 | .112 | .115 |
| likes_preschool | .02 | .13 | 0-1 | -.028 | 1.000 |
| likes_printingservice | .02 | .17 | 0-2 | .015 | 1.000 |
| likes_privateschool | .02 | .15 | 0-1 | .072 | 1.000 |
| likes_producer | .09 | 1.44 | 0-47 | .096 | .798 |
| likes_productservice | .93 | 1.83 | 0-21 | .108 | .191 |
| likes_professionalgamer | .01 | .12 | 0-1 | .057 | 1.000 |
| likes_professionalservice | .07 | .33 | 0-5 | .045 | 1.000 |
| likes_psychologist | .20 | 1.18 | 0-24 | .022 | 1.000 |
| likes_psychotherapist | .03 | .20 | 0-4 | -.013 | 1.000 |
| likes_pub | .20 | .62 | 0-6 | .084 | 1.000 |
| likes_publicfigure | 3.01 | 6.69 | 0-132 | .220 | <.001 |
| likes_publicgovernmentservic | .07 | .29 | 0-4 | .068 | 1.000 |
| likes_publicrelationsagency | .01 | .14 | 0-3 | .087 | 1.000 |
| likes_publicservice | .02 | .16 | 0-2 | .054 | 1.000 |
| likes_publicswimmingpool | .02 | .16 | 0-2 | .011 | 1.000 |
| likes_publisher | .51 | 2.58 | 0-57 | .122 | .027 |
| likes_radiostation | .15 | .51 | 0-5 | .080 | 1.000 |
| likes_realestate | .03 | .21 | 0-5 | .045 | 1.000 |
| likes_realestateagent | .02 | .18 | 0-2 | -.016 | 1.000 |
| likes_realestateservice | .03 | .20 | 0-2 | .086 | 1.000 |
| likes_recordlabel | .14 | .57 | 0-8 | .128 | .012 |
| likes_recreationcenter | .02 | .16 | 0-3 | .024 | 1.000 |
| likes_recreationsportswebsit | .06 | .30 | 0-4 | .022 | 1.000 |
| likes_referencewebsite | .12 | .41 | 0-4 | .041 | 1.000 |
| likes_region | .03 | .17 | 0-2 | .065 | 1.000 |
| likes_regionalwebsite | .02 | .16 | 0-3 | -.026 | 1.000 |
| likes_religiousorganization | .15 | .74 | 0-15 | .101 | .433 |
| likes_religiousplaceofworship | .01 | .11 | 0-1 | .068 | 1.000 |
| likes_residence | .01 | .13 | 0-2 | .001 | 1.000 |
| likes_restaurant | .59 | 1.51 | 0-19 | .101 | .442 |
| likes_retailcompany | .19 | .56 | 0-5 | .137 | .003 |
| likes_salsaclub | .01 | .12 | 0-2 | .010 | 1.000 |
| likes_sandwichshop | .03 | .22 | 0-4 | .050 | 1.000 |
| likes_school | .24 | .67 | 0-5 | .082 | 1.000 |
| likes_schoolsportsteam | .02 | .15 | 0-2 | .043 | 1.000 |
| likes_science | .02 | .15 | 0-1 | .031 | 1.000 |
| likes_sciencemuseum | .03 | .16 | 0-1 | -.006 | 1.000 |
| likes_sciencetechnologyengi | .05 | .32 | 0-4 | .034 | 1.000 |
| likes_sciencewebsite | .26 | .90 | 0-18 | .099 | .549 |
| likes_scientist | .10 | .37 | 0-5 | .062 | 1.000 |
| likes_seafoodrestaurant | .03 | .22 | 0-5 | .028 | 1.000 |
| likes_shoppingmall | .07 | .32 | 0-3 | .086 | 1.000 |
| likes_shoppingretail | .36 | .76 | 0-6 | .089 | 1.000 |
| likes_shoppingservice | .01 | .10 | 0-1 | .012 | 1.000 |
| likes_show | .13 | .51 | 0-8 | .077 | 1.000 |
| likes_skincareservice | .02 | .12 | 0-1 | .080 | 1.000 |
| likes_skiresort | .02 | .14 | 0-2 | .078 | 1.000 |
| likes_socialclub | .08 | .32 | 0-3 | .087 | 1.000 |
| likes_socialmediaagency | .03 | .19 | 0-3 | .113 | .094 |
| likes_socialservice | .01 | .12 | 0-2 | .030 | 1.000 |
| likes_societyculturewebsite | .38 | .91 | 0-7 | .083 | 1.000 |
| likes_software | .07 | .36 | 0-5 | .072 | 1.000 |
| likes_softwarecompany | .01 | .12 | 0-2 | .046 | 1.000 |
| likes_song | .03 | .17 | 0-2 | .051 | 1.000 |
| likes_spanishrestaurant | .01 | .12 | 0-1 | .061 | 1.000 |
| likes_specialtygrocerystore | .07 | .32 | 0-4 | .077 | 1.000 |
| likes_specialtyschool | .02 | .13 | 0-2 | -.007 | 1.000 |
| likes_sportinggoodsstore | .04 | .25 | 0-3 | .040 | 1.000 |
| likes_sports | .07 | .30 | 0-3 | .024 | 1.000 |
| likes_sportsclub | .07 | .32 | 0-4 | .053 | 1.000 |
| likes_sportsevent | .08 | .37 | 0-6 | .084 | 1.000 |
| likes_sportsfitnessinstructi | .01 | .12 | 0-1 | .085 | 1.000 |
| likes_sportsleague | .14 | .51 | 0-7 | .022 | 1.000 |
| likes_sportsrecreation | .22 | .65 | 0-5 | .021 | 1.000 |
| likes_sportsteam | .32 | 1.16 | 0-22 | .062 | 1.000 |
| likes_sportswearstore | .02 | .16 | 0-2 | .077 | 1.000 |
| likes_stadiumarenasportsve | .07 | .31 | 0-3 | .061 | 1.000 |
| likes_steakhouse | .01 | .14 | 0-3 | .041 | 1.000 |
| likes_street | .01 | .11 | 0-1 | .112 | .103 |
| likes_sunglasseseyewearstore | .02 | .15 | 0-2 | .021 | 1.000 |
| likes_sushirestaurant | .05 | .27 | 0-3 | .081 | 1.000 |
| likes_tapasbarrestaurant | .02 | .12 | 0-1 | -.007 | 1.000 |
| likes_tattoopiercingshop | .13 | .56 | 0-8 | .062 | 1.000 |
| likes_teacher | .04 | .21 | 0-2 | .027 | 1.000 |
| likes_tearoom | .04 | .21 | 0-2 | .054 | 1.000 |
| likes_telecommunicationcompany | .05 | .23 | 0-2 | .108 | .181 |
| likes_theatricalplay | .02 | .17 | 0-3 | .065 | 1.000 |
| likes_theatricalproductions | .02 | .17 | 0-3 | .043 | 1.000 |
| likes_themerestaurant | .01 | .12 | 0-2 | .002 | 1.000 |
| likes_toolsequipment | .02 | .13 | 0-2 | .004 | 1.000 |
| likes_touragency | .12 | .49 | 0-7 | .127 | .014 |
| likes_tourguide | .04 | .22 | 0-2 | -.001 | 1.000 |
| likes_touristinformationcenter | .07 | .32 | 0-4 | .060 | 1.000 |
| likes_toystore | .02 | .15 | 0-3 | -.003 | 1.000 |
| likes_transitstop | .02 | .13 | 0-2 | .021 | 1.000 |
| likes_transitsystem | .02 | .18 | 0-4 | .077 | 1.000 |
| likes_translator | .03 | .79 | 0-26 | .071 | 1.000 |
| likes_transportationservice | .07 | .34 | 0-3 | .085 | 1.000 |
| likes_travelagency | .12 | .50 | 0-6 | .078 | 1.000 |
| likes_travelcompany | .20 | .66 | 0-7 | .066 | 1.000 |
| likes_travelservice | .03 | .17 | 0-2 | .079 | 1.000 |
| likes_traveltransportation | .02 | .13 | 0-1 | .009 | 1.000 |
| likes_tutorteacher | .01 | .12 | 0-1 | -.047 | 1.000 |
| likes_tvchannel | .38 | .83 | 0-13 | .172 | <.001 |
| likes_tvmovieaward | .06 | .39 | 0-7 | .045 | 1.000 |
| likes_tvmovies | .03 | .22 | 0-5 | .095 | .863 |
| likes_tvnetwork | .12 | .42 | 0-6 | .161 | <.001 |
| likes_tvseason | .03 | .19 | 0-3 | .104 | .299 |
| likes_tvshow | 1.50 | 3.35 | 0-63 | .169 | <.001 |
| likes_vacationhomerental | .02 | .16 | 0-2 | -.001 | 1.000 |
| likes_vegetarianveganrestauran | .02 | .18 | 0-3 | -.050 | 1.000 |
| likes_veterinarian | .03 | .22 | 0-5 | .014 | 1.000 |
| likes_video | .04 | .20 | 0-2 | .077 | 1.000 |
| likes_videocreator | .17 | .47 | 0-4 | .118 | .050 |
| likes_videogame | .20 | 1.17 | 0-28 | -.001 | 1.000 |
| likes_vintagestore | .03 | .21 | 0-3 | .042 | 1.000 |
| likes_visualarts | .22 | .65 | 0-12 | .125 | .018 |
| likes_vitaminssupplements | .04 | .30 | 0-6 | .109 | .150 |
| likes_waterpark | .01 | .12 | 0-2 | .067 | 1.000 |
| likes_webdesigner | .03 | .29 | 0-5 | .070 | 1.000 |
| likes_website | 1.15 | 2.22 | 0-21 | .165 | <.001 |
| likes_weddingplanningservice | .05 | .30 | 0-6 | .044 | 1.000 |
| likes_wholesalesupplystore | .02 | .17 | 0-2 | .047 | 1.000 |
| likes_wildlifesanctuary | .01 | .10 | 0-1 | -.007 | 1.000 |
| likes_winebar | .07 | .39 | 0-9 | .060 | 1.000 |
| likes_winebeerspiritsstore | .02 | .13 | 0-2 | .040 | 1.000 |
| likes_wineryvineyard | .04 | .31 | 0-6 | .035 | 1.000 |
| likes_winespirits | .11 | .71 | 0-20 | .052 | 1.000 |
| likes_womensclothingstore | .11 | .42 | 0-5 | .050 | 1.000 |
| likes_workplaceoffice | .02 | .13 | 0-1 | .041 | 1.000 |
| likes_writer | .47 | 1.45 | 0-36 | .154 | <.001 |
| likes_yogastudio | .02 | .26 | 0-7 | .003 | 1.000 |
| likes_youthorganization | .09 | .37 | 0-4 | .047 | 1.000 |
| likes_zoo | .01 | .10 | 0-1 | .012 | 1.000 |
